# The dormant blood microbiome in chronic, inflammatory diseases

**DOI:** 10.1093/femsre/fuv013

**Published:** 2015-05-04

**Authors:** Marnie Potgieter, Janette Bester, Douglas B. Kell, Etheresia Pretorius

**Affiliations:** 1 Department of Physiology, Faculty of Health Sciences, University of Pretoria, Arcadia 0007, South Africa; 2 School of Chemistry and The Manchester Institute of Biotechnology, The University of Manchester, 131, Princess St, Manchester M1 7DN, Lancs, UK

**Keywords:** ‘sterile’ blood microbiome, culturability, dormancy, dysbiosis, atopobiosis, Parkinson's disease, Alzheimer disease

## Abstract

Blood in healthy organisms is seen as a ‘sterile’ environment: it lacks proliferating microbes. Dormant or not-immediately-culturable forms are not absent, however, as intracellular dormancy is well established. We highlight here that a great many pathogens can survive in blood and inside erythrocytes. ‘Non-culturability’, reflected by discrepancies between plate counts and total counts, is commonplace in environmental microbiology. It is overcome by improved culturing methods, and we asked how common this would be in blood. A number of recent, sequence-based and ultramicroscopic studies have uncovered an authentic blood microbiome in a number of non-communicable diseases. The chief origin of these microbes is the gut microbiome (especially when it shifts composition to a pathogenic state, known as ‘dysbiosis’). Another source is microbes translocated from the oral cavity. ‘Dysbiosis’ is also used to describe translocation of cells into blood or other tissues. To avoid ambiguity, we here use the term ‘atopobiosis’ for microbes that appear in places other than their normal location. Atopobiosis may contribute to the dynamics of a variety of inflammatory diseases. Overall, it seems that many more chronic, non-communicable, inflammatory diseases may have a microbial component than are presently considered, and may be treatable using bactericidal antibiotics or vaccines.

## Abstract (precis)

Atopobiosis of microbes (the term describing microbes that appear in places other than where they should be), as well as the products of their metabolism, seems to correlate with, and may contribute to, the dynamics of a variety of inflammatory diseases.

## Graphical abstract

**Graphical Abstract Figure.**
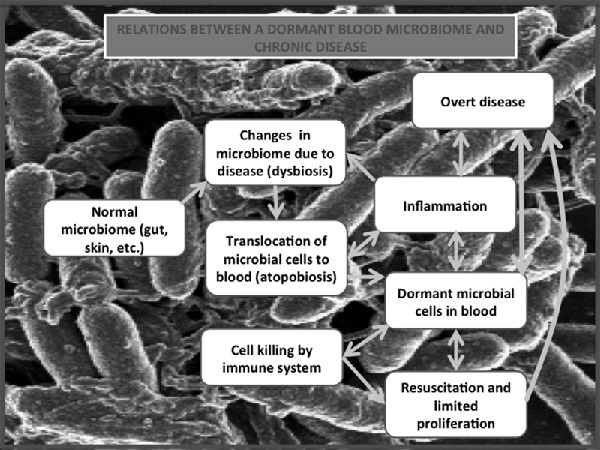
Atopobiosis of microbes (the term describing microbes that appear in places other than where they should be), as well as the products of their metabolism, seems to correlate with, and may contribute to, the dynamics of a variety of inflammatory diseases.

## INTRODUCTION

‘Overall, it seems inevitable that the availability of these methods will cause the catalog of disease states recognized as having a microbial contribution to their etiology to expand enormously in the short term, particularly as improved methods for resuscitation of small cell numbers are found’ (Davey and Kell 1996).

**Figure 1.**
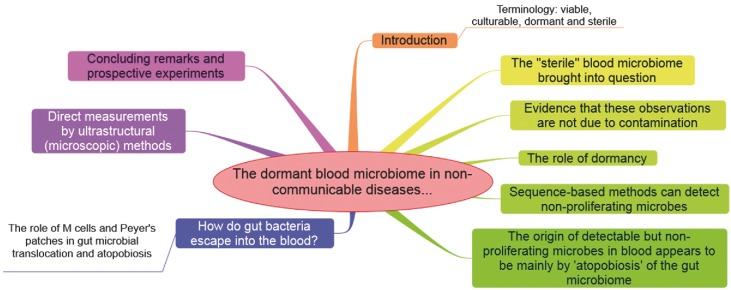
An overview figure summarizing the contents of this manuscript.

Over the years, a variety of diseases that were previously considered non-communicable have been found to have a microbial component, the role of *Helicobacter pylori* in ulcerogenesis (Marshall and Warren 1984) being a particularly well-known example. There have also been hints for a microbial component to many other non-communicable diseases, but culturing the relevant organisms has rarely been successful. However, there is increasing recognition that microbes may be present in forms that are not easily culturable, and a number of recent articles have brought these possibilities more sharply into focus. Our aim is to review these developments. The manuscript structure is shown in Fig. 1.

### A note on terminology: viable, culturable, dormant and sterile

In this field, much confusion has arisen historically because of a failure to recognize that most microbes reproduce by binary fission and that this reproduction must be a minimal property or hallmark of a microbial cell that possesses ‘life’ or is ‘alive’ (Proal, Albert and Marshall 2011). Thus, as with Schrödinger's cat (e.g. Primas 1981; Gribbin 1985), we cannot say that an individual microbial cell ‘is’ alive, only (if true) that it ‘was’ alive, since it will by then have become two cells. This implies that being alive is not best treated as though it were an innate property of a cell, but the definition must be operational, and include both the cell and the ‘environment’ (experiment) used to detect the status *a posteriori* (Kell *et al.* 1998).

Thus, as with Postgate (e.g. Postgate 1967, 1969, 1976), we equate viability with culturability, and stress that culturability—the ability to reproduce—is to be determined operationally. Other methods that do not determine culturability are not tests of viability per se, but merely measure what they measure (e.g. the content of a chemical such as ATP, membrane permeability to a dye, enzymatic activity, macromolecular sequences and so on). In addition, it is impossible in principle to (cor)relate macroscopic measurements of a culture with the ability of individual cells to divide (Kell *et al.* 1991; Davey and Kell 1996). In other words, if the macroscopic ATP content of say a starving culture were to decrease by 50%, we would not know if all of the cells had lost half their ATP or half of the cells had lost all of their ATP (or anything in between). The culturability of the former would likely be 50% and of the latter 100%, despite the same macroscopic ATP content.

**Figure 2.**
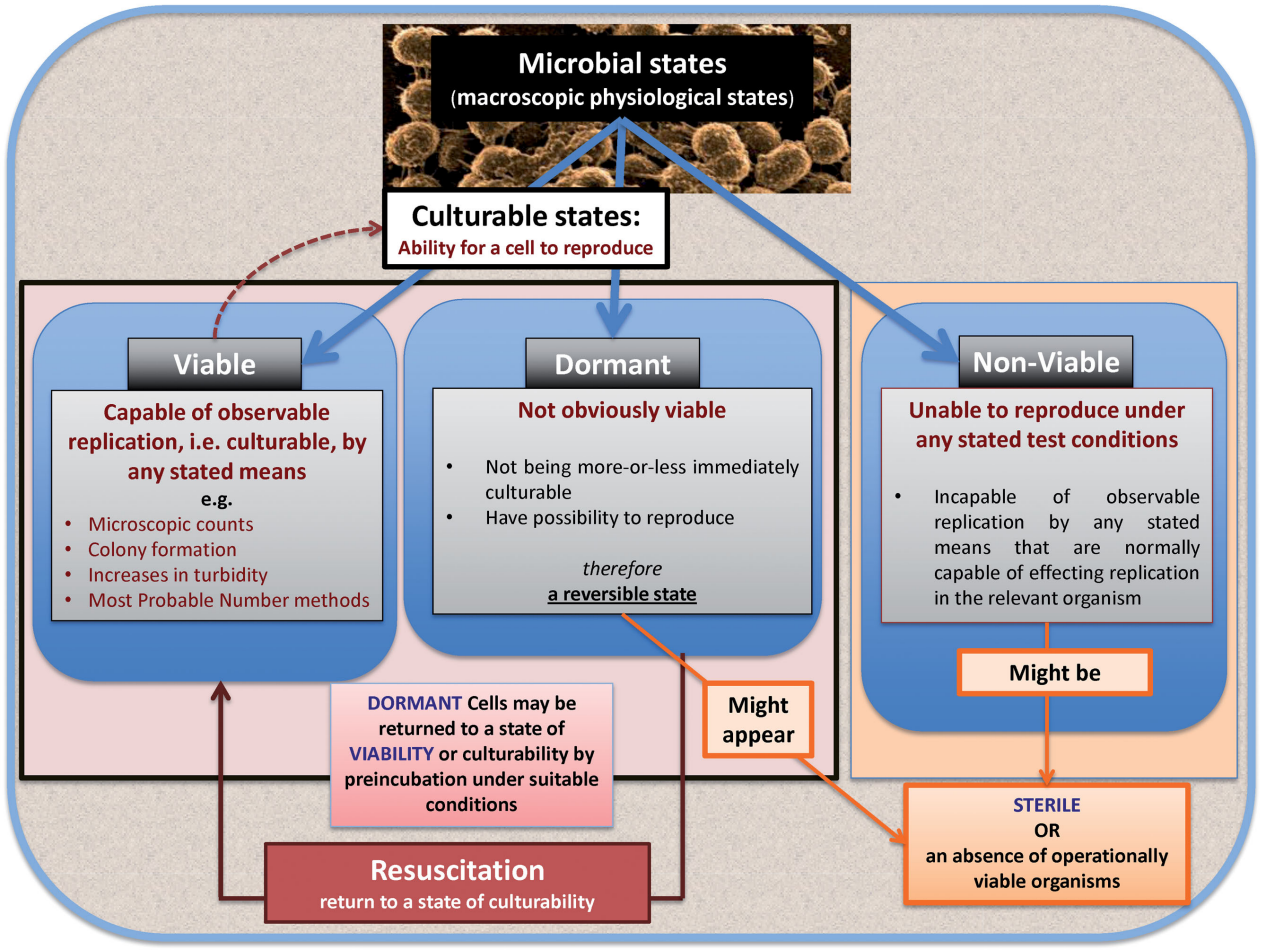
A diagrammatic representation of the major macroscopic physiological states of microbes and their interrelationships.

A lack of culturability may mean that a cell is non-viable under the circumstances tested, but viability or non-viability are not the only two possible states here. An apparent non-culturability of a surviving cell also admits another possibility, for which the natural term is ‘dormant’ (Kaprelyants, Gottschal and Kell 1993; Epstein 2013). This is that the cell is not presently culturable (viable), but it is not ‘dead’ (in the sense of an operationally irreversible loss of viability) in that it may be induced to return to a state of culturability (by a process or processes typically referred to as ‘resuscitation’). This also means that the term ‘viable-but-non-culturable’, while quite common in use, is in fact an oxymoron that is to be discouraged (Kell *et al.* 1998). The eminent microbial physiologist Howard Gest is similarly scathing about the term ‘unculturable’ (Gest 2008), noting that one just needs to try harder to culture organisms. Table 1 shows the three terms best suited to discuss these issues, while Fig. 2 shows a diagrammatic representation of the macroscopic physiological microbial states we mostly consider.

**Table 1.** Operational definitions of viable, non-viable and dormant microbes. These are the three terms we consider best suited to describe the macroscopic physiological states of microbes as regards their ability to replicate. We note that the terms ‘not immediately culturable’ (NIC) and ‘active but not culturable’ (ABNC) can also have some utility (Kell *et al.* 1998), while dormant cells are sometimes referred to as ‘persisters’. Other variants of ‘dormancy’ that have been used include ‘anabiosis’ (Keilin 1959) and ‘cryptobiosis’ (e.g. Clegg 2001; Neuman 2006); all these terms imply a reversible state between the appearance of being living and non-living in different circumstances. This definition of dormancy also likely includes cells that may operationally be ‘injured’, and possibly wall-less L-forms (Domingue and Woody 1997; Mattman 2001; Allan, Hoischen and Gumpert 2009; Domínguez-Cuevas *et al.* 2012; Errington 2013; Mercier, Kawai and Errington 2013, 2014) provided they are or may become culturable. ‘Sterile’ refers to an absence of operationally viable organisms as defined in this table.

**Term**	**Properties**
Viable	Capable of observable replication, i.e. culturable, by any stated means.
Non-viable	Incapable of observable replication by any stated means normally capable of effecting replication in the relevant organism.
Dormant	Not viable in the sense of not being more or less immediately culturable, but may be returned to a state of viability or culturability by preincubation under suitable conditions.

The assessment of replication potential (culturability) of individual cells may be done microscopically (e.g. by microscopic counts) or macroscopically (e.g. via colony formation on an agar plate or through the ‘most probable number’ technique). The latter has the advantage of potentially assessing dormancy in the absence of any contaminating culturable cells that might proliferate during the assay (Kaprelyants, Mukamolova and Kell 1994; Votyakova, Kaprelyants and Kell 1994; Kell *et al.* 1998). For assessing culturability (=viability), we do not therefore include other strategies in which cells do not actually divide, such as the so-called direct viable count of Kogure, Simidu and Taga (1979). Thus, we here highlight the point that the possibility of microbial dormancy means that a system that appears to be devoid of culturable microbes may still contain dormant cells or forms that may become culturable.

### The ‘sterile’ blood microbiome brought into question

The circulation is a closed system and the blood in healthy organisms was first believed to be a sterile environment (Drennan 1942; Proal, Albert and Marshall 2014). This definition is used in the most usual sense of an absence of culturable microbes, since blood can of course provide a suitable growth medium for microbes (as in blood culture; Wilson and Weinstein 1994; Weinstein 1996; Schroeter *et al.* 2012; cf. Valencia-Shelton and Loeffelholz 2014), and any bacteraemia or sepsis, even at 1–10 cells mL^−1^ (Murray 2015), is potentially life-threatening (e.g. Vincent *et al.* 2009; Eleftheriadis *et al.* 2011; Havey, Fowler and Daneman 2011; Montassier *et al.* 2013). However, the principle of the presence of truly sterile blood in healthy humans has been challenged, as operationally it does not mean that dormant or non-culturable forms of organisms are absent (Kaprelyants, Gottschal and Kell 1993; Kell *et al.* 1998; McLaughlin *et al.* 2002) (see Table 1). Nearly 50 years ago, the existence of a novel bacteriological system was noted in 71% of blood samples taken from diseased humans and from 7% of supposedly healthy humans, when RBCs were lysed (Domingue and Schlegel 1977). A year later, corynebacteria-like microorganisms developing in hemocultures were shown within RBCs (Tedeschi *et al.* 1978), and in 2001 it was found that even ‘healthy’ blood specimens can contain bacterial 16S ribosomal DNA (Nikkari *et al.* 2001). Domingue and Woody (1997) and Domingue (2010) summarizes much of this earlier literature. L-forms are bacterial variants that lack some or all of a cell wall. Nonetheless they can divide, especially in osmotically stabilized media, by processes that variously involve membrane blebbing, tubulation, vesiculation and fission (Allan, Hoischen and Gumpert 2009; Errington 2013; Mercier, Kawai and Errington 2014). While it remains unclear whether what was seen in these earlier studies (Domingue and Woody 1997; Domingue 2010) may have been L-forms (Mattman 2001), that could in time revert to normal bacteria under the correct conditions (Casadesús 2007), L-forms are becoming a topic of considerable current research (Devine 2012; Domínguez-Cuevas *et al.* 2012; Mercier, Kawai and Errington 2013, 2014).

The presence of a blood bacterial microbiome has also been associated with a variety of infectious, as well as non-infectious disease states (Huang *et al.* 2006; Thwaites and Gant 2011; Nielsen *et al.* 2012; Prajsnar *et al.* 2012; Wang *et al.* 2012a; Kibru *et al.* 2014; Sato *et al.* 2014). It is, for example, known that *H. pylori* can exist not only in the gastric mucosa but also in peripheral blood, where it could cause bacteremia (Huang *et al.* 2006), and could contribute to Parkinson's disease (PD) or related pathologies that precede motor symptoms (Nielsen *et al.* 2012). *Helicobacter pylori* was also previously implicated in the development of anemia (Wang *et al.* 2012b; Kibru *et al.* 2014). *Staphylococcus aureus* can also use neutrophils as ‘Trojan horses’ to disseminate infection (Thwaites and Gant 2011; Prajsnar *et al.* 2012), while many other pathogens, such as *Listeria monocytogenes* (Xayarath and Freitag 2012), *Salmonella typhimurium* (Eisenreich *et al.* 2010; Claudi *et al.* 2014; Holden 2015) and *Yersinia pestis* (Isberg 1991), are well known to persist intracellularly; Gest (2008) gives other historical examples. The same is true for viruses, which are not discussed here.

The presence of an aberrant blood microbiota (as assessed by sequencing) has been implicated in type II diabetes and cardiovascular disease (Amar *et al.* 2011, 2013; Sato *et al.* 2014). There is also growing evidence that periodontal disease and gingivitis are closely linked to cardiovascular disease (Yang *et al.* 2013; Ramírez *et al.* 2014). Oral bacterial translocation into the blood has been implicated in the development of periodontal disease-induced endocarditis and myocardial and/or cerebral infarction, especially in patients with heart valve dysfunction (Koren *et al.* 2011; Amar and Engelke 2014; Seringec *et al.* 2014).

We will argue in the next sections that the existence of potentially viable (but possibly non-proliferating) pleomorphic bacteria in the blood of healthy humans (McLaughlin *et al.* 2002) may therefore be of some significance in pathology. If such a microbiome can disrupt homeostasis, it can ultimately play a fundamental role in disease development and progression. It has therefore been proposed that the blood microbiota might therefore represent or contribute to the first step in the kinetics of atherosclerosis (Sato *et al.* 2014), cardiovascular disease and type II diabetes (Amar *et al.* 2011), and therefore ultimately serve as biomarkers for cardiovascular disease risk (Amar *et al.* 2013). However, in the quest to use the blood microbiota as biomarkers, the question of detectability and cultivability are key concepts.

In particular, the existence of a blood microbiome is only really meaningful and of scientific interest if it represents an undisturbed state, and is not, for instance, an artefact caused by the external introduction of microbes through human intervention, reagent contamination (Schroeter *et al.* 2012; Salter *et al.* 2014) and so forth. We therefore rehearse the evidence that while such artefacts are certainly possible, and must be excluded rigorously, the phenomenon of a human blood microbiome cannot be dismissed as such an artefact *in toto*.

### Evidence that these observations are not due to contamination

While contamination from reagents (e.g. Schroeter *et al.* 2012; Salter *et al.* 2014), or simply poor sterile technique with needles and so on, can lead to an artefactual appearance of a blood microbiome, we consider that the following arguments, taken together, exclude the thought that the entire (and considerable) literature on a blood microbiome can be explained via contamination.

- The first argument is that there are significant differences between the blood microbiomes of individuals harboring disease states and nominally healthy controls, despite the fact that samples are treated identically (see later). Some similar arguments apply to the assessment of drug transporters under different conditions (Kell and Oliver 2014).
- A second argument is that the morphological type of organism (e.g. coccus versus bacillus) seems to be characteristic of particular diseases.
- A third argument is that in many cases (see below) relevant organisms lurk intracellularly, which is hard to explain by contamination.
- A fourth argument is that there are just too many diseases where bacteria have been found to play a role in the pathogenesis, that all of them may be caused by contamination.
- Finally, the actual numbers of cells involved seem far too great to be explicable by contamination; given that blood contains more than 10^9^ erythrocytes mL^−1^, if there was just one bacterial cell per 100 000 erythrocytes (see below and Amar *et al.* 2011), this will equate to 10^4^ bacteria mL^−1^. These are not small numbers.

It is important to point out that molecular methods have been used frequently to detect active sepsis. These selfsame methods are also used in environmental biology (as we pointed out in this review), without undue concern about the potential for contamination. Contamination will always be a concern, of course, as noted by Nikkari *et al.* (2001), but many papers since 2001 have documented strategies for detecting prokaryotic DNA in blood and serum using appropriate and careful controls (Anthony *et al.* 2000; Mylotte and Tayara 2000; Jiang *et al.* 2009; Varani *et al.* 2009; Mancini *et al.* 2010; Chang *et al.* 2011; Grif *et al.* 2012a; Fernández-Cruz *et al.* 2013; Gaibani *et al.* 2013). Also, detecting bacteria in blood cultures during sepsis is considered the standard diagnostic tool for blood stream infections (Muñoz *et al.* 2008; Varani *et al.* 2009), and some laboratories consider that e.g. PCR testing should always be a complement for the traditional blood culture test (Grif *et al.* 2012b).

### The role of dormancy

Dormancy in microbiology is of course well known, even for non-sporulating bacteria, and has been defined as a stable but reversible nonreplicating state (Mariotti *et al.* 2013; see also Table 1 and Kaprelyants, Gottschal and Kell 1993; Kell *et al.* 1998, 2003). The importance of dormant or non-cultured (as opposed to ‘non-culturable’) organisms has long been recognized in environmental microbiology (e.g. Mason, Hamer and Bryers 1986; Amann, Ludwig and Schleifer 1995; Eilers *et al.* 2000; Hugenholtz 2002; Keller and Zengler 2004; Pham and Kim 2012; Epstein 2013), because of the 100-fold or greater difference between microscopically observable cells and those capable of forming a colony on an agar plate (‘the great plate count anomaly’, see below).

Of the four main possibilities, what we do not know in general is whether the ‘missing’ cells

- are incapable of growth on the enrichment/isolation media,
- are killed by the enrichment/isolation media (e.g. Tanaka *et al.* 2014),
- have lost viability irreversibly (i.e. are operationally dead) or
- are in a dormant or not-immediately-culturable state from which we might resuscitate them (to effect culturability) if only we knew how.

The fact that typical isolation media and incubation conditions do not admit the measurable growth of all strains is certainly well known (indeed it is the basis for selective isolation media!), and it took a good while to learn how to culture pathogens such as *H. pylori* (Marshall and Warren 1984; Marshall 2006), *Legionella pneumophila* (Feeley *et al.* 1978; Saito *et al.* 1981; Meyer 1983), *Tropheryma* whipplei (Maiwald and Relman 2001; Maiwald *et al.* 2003; Renesto *et al.* 2003) and so on (Singh *et al.* 2013). The majority of bacteria that persist in a ‘non-culturable’ form in wounds (e.g. Dowd *et al.* 2008; Percival *et al.* 2012), or in diseases such as cystic fibrosis (Lewis 2010) or tuberculosis (Young, Stark and Kirschner 2008; Zhang, Yew and Barer 2012), and even simply in conventional cultures of *Escherichia coli* (e.g. Koch 1987; Balaban *et al.* 2004; Keren *et al.* 2004a,b; Gerdes and Maisonneuve 2012; Amato, Orman and Brynildsen 2013; Germain *et al.* 2013; Maisonneuve, Castro-Camargo and Gerdes 2013; Maisonneuve and Gerdes 2014; Holden 2015), where phenotypic culture differentiation is well established (Koch 1971), are also ‘normally culturable’ by established means. Thus, the existence of operationally ‘non-culturable’ forms of only moderately fastidious bacteria is very well established, and more and more bacteria previously thought ‘unculturable’ are being brought into culture (e.g. Zengler *et al.* 2002; Keller and Zengler 2004; Stevenson *et al.* 2004; Gich *et al.* 2005; Kamagata and Tamaki 2005; D'Onofrio *et al.* 2010; Nichols *et al.* 2010; Vartoukian, Palmer and Wade 2010; Dedysh 2011; Pham and Kim 2012; Puspita *et al.* 2012, 2013; Stewart 2012; Allen-Vercoe 2013; Narihiro and Kamagata 2013; Singh *et al.* 2013; Walker *et al.* 2014; Lagier *et al.* 2015a,b; Ling *et al.* 2015).

In environmental microbiology, some bacteria pass through the usual 0.2 μm filters, and have been referred to as ‘ultramicrobacteria’ (Macdonell and Hood 1982; Morita 1997). It was proposed (Kaprelyants, Gottschal and Kell 1993) that rather than being small (starved) forms of normal bacteria they were more likely to be normal forms of small bacteria, and this seems to have been accepted (Lysak *et al.* 2010; Sahin *et al.* 2010; Duda *et al.* 2012; Soina *et al.* 2012).

The ability to culture certain kinds of soil bacteria by preincubation in weak broth is also well established (e.g. Bakken and Olsen 1987; Kaprelyants, Gottschal and Kell 1993), and our own experiments showed very high levels of resuscitability of dormant cells of *Micrococcus luteus* (e.g. Kaprelyants and Kell 1993; Kaprelyants, Mukamolova and Kell 1994; Kaprelyants *et al.* 1996, 1999; Kell *et al.* 1998, 2003; Mukamolova *et al.* 1998a,b, 1999, 2002a,b). In a similar way, substrate-accelerated death of non- or slowly growing microorganisms has been known for decades (Postgate 1967; Calcott and Postgate 1972; Calcott and Calvert 1981).

Thus, any of several well-established mechanisms may contribute to the (often) large differences observable between microscopic counts and the number of operationally culturable microbes, with the greatest likelihood being that we simply have to develop more and better methods to bring these strains back into culture, i.e. to resuscitate them. In particular, however, this ‘great plate count anomaly’ has, of course, been brought into much sharper focus because of the advent of culture-independent, sequence-based means for detecting and (to a certain extent) enumerating microbes (though not, of course, of assessing their culturability).

### Sequence-based methods for detecting non-proliferating microbes

The vast majority of microbial species remain uncultivated and, until recently, about half of all known bacterial phyla were identified only from their 16S ribosomal RNA gene sequence (Lasken and McLean 2014). Also, single-cell genomics is a powerful tool for accessing genetic information from uncultivated microorganisms (Lasken 2012; Rinke *et al.* 2013; Cavanagh *et al.* 2014; Clingenpeel *et al.* 2014). Bacterial single-cell genome sequencing and bioinformatics are, however, challenging (Pallen, Loman and Penn 2010; Didelot *et al.* 2012; Loman *et al.* 2012; Fricke and Rasko 2014).

The development of sequence-based methods for microbes (and especially non-eukaryotes) owes much to the pioneering work of Carl Woese and colleagues, who recognized the utility of small subunit ribosomal RNA (based on both its essentiality and the small but significant sequence variations) and applied it with great effect in molecular phylogenetics (Woese and Fox 1977; Woese, Kandler and Wheelis 1990). Notwithstanding modern reinterpretations of the taxonomic details derived therefrom (e.g. Williams *et al.* 2013), there can be little doubt that this work drew the attention of microbiologists to the potential of sequence-based methods for detecting microbes that were then invisible to methods based solely on culture, e.g. in clinical microbiology (Didelot *et al.* 2012; Loman *et al.* 2012; Proal *et al.* 2013; Fricke and Rasko 2014). rRNA remains a widely used strategy for detecting specific microbes. This has of course led to metagenomics, the large-scale sequencing of macromolecules and indeed (statistically) entire genomes from complex (non-axenic) environments, increasing the requirement for a full set of complete reference sequences (Kyrpides *et al.* 2014) and not just those of 16S rRNA (Yarza *et al.* 2013). Even the coupling of sequences to activities has now become possible (e.g. Radajewski *et al.* 2000; Wang *et al.* 2012c).

### Microbiome analyses: latest technologies employed

More recently, gut metagenomics has been systematized with NIH's Human Microbiome project (HMP) and the European MetaHIT project aiming to deciphering the structure and function of the human gut microbiota (Fredricks 2013; Robles-Alonso and Guarner 2014). The HMP has developed a reference collection of 16S ribosomal RNA gene sequences collected from sites across the human body (Koren *et al.* 2013; Ding and Schloss 2014). This information can be used to associate changes in the microbiome with changes in health, and particularly also the blood microbiome. The Integrative Human Microbiome Project (iHMP, http://hmp2.org), the second phase of the NIH HMP, aims to study the interactions by analyzing microbiome and host activities in longitudinal studies of disease-specific cohorts and by creating integrated data sets of microbiome and host functional properties (The Integrative HMP (iHMP) Research Network Consortium 2014), ultimately allowing us to analyze host and microbial DNA (genome) and RNA (transcriptome) sequences (Morgan and Huttenhower 2014). However, in the HMP study, the main anatomic sites where samples are collected are skin, mouth, nose, colon and vagina (ElRakaiby *et al.* 2014). So far as we are aware, these projects do not focus on the blood microbiome (which is probably unsurprising when most commentators assume that it does not exist).

The gut microbiome is by far the largest numerically, and our purpose here is not to review it in any detail, since this has been done very well in terms of

- its constitution (Lozupone *et al.* 2012; Weinstock 2012),
- temporal variation (Caporaso *et al.* 2011; Flores *et al.* 2014; Thaiss *et al.* 2014),
- changes associated with diet (Muegge *et al.* 2011),
- obesity (Turnbaugh *et al.* 2006, 2009),
- age and geography (Delzenne and Cani 2011; Delzenne *et al.* 2011; Yatsunenko *et al.* 2012),
- inflammation (Cani *et al.* 2008, 2012),
- the immune system (Kau *et al.* 2011; McDermott and Huffnagle 2014)
- and various pathologies (Pflughoeft and Versalovic 2012; Schulz *et al.* 2014).

It was implied that a better understanding of microbiome-encoded pathways for xenobiotic metabolism might also have implications for improving the efficacy of pharmacologic interventions with neuromodulatory agents (Gonzalez *et al.* 2011), and that the exploration of microbiome and metagenome might give us insightful new perspectives regarding human genetics and how the microbiota contribute to immunity, as well as to metabolic and inflammatory diseases (Cho and Blaser 2012; Blaser *et al.* 2013; Blaser 2014; Leslie and Young 2015). This is because it is assumed in such studies that it is the small-molecule products of the gut microbiome that can appear in the human serum metabolome, and thus influence the rest of the human body (e.g. Wikoff *et al.* 2009; Holmes *et al.* 2011; Le Chatelier *et al.* 2013, and see Table 2). Here we also need to mention lipopolysaccharide (LPS), a main constituent of the Gram-negative outer membrane that induces the production of cytokines and/or chemokines, which in turn regulate inflammatory and innate and subsequent adaptive immune responses (Glaros *et al.* 2013; Rhee 2014; Ronco 2014). The release of LPS may therefore change gut homeostasis, may play a role in e.g. inflammatory bowel disease and necrotizing enterocolitis (Rhee 2014), and may certainly act as an acute phase protein in sepsis (Ding and Jin 2014).

**Table 2.** Some examples of small molecule gut metabolites whose secretion has been implicated in various disease states.

Metabolite	Intermediates/products	Synthesis	Role in health and disease	References
Amino acids		The gut microbiota is not itself an important source of amino acids during periods of adequate protein intake. Some commensal members produce biologically active components from amino acids. Amino acid supplementation in a mouse model of ulcerative colitis has been shown to promote overall growth of commensal microbiota. The effect was considered to be mediated via the stimulatory effect on mucin production by amino acid supplementation.		Faure *et al.* (2006); Devaraj, Hemarajata and Versalovic (2013); Bergen (2014)
Benzoates	Benzoic acid, hippurate, 2-hydroxyhippurate	Gut microbiota in mice with active colitis displayed enrichment for genes involved in benzoate degradation. Hippurate derives from plant food polyphenols and is a conjugate of benzoic acid with glycine. In humans a large portion of hippurate is believed to be derived from precursors absorbed in the small intestines. It is reliably decreased in IBD.		Rechner *et al.* (2002); Aronov *et al.* (2011); De Preter and Verbeke (2013); Rooks *et al.* (2014)
Bile acids		Bile acids are synthesized from cholesterol in the liver and further metabolized into secondary bile acids by the gut microbiota. The amino acid sides chain of glyco- and tauro-conjugated bile acids are cleaved by bacterial bile salt hydrolase (BSH) enzyme to yield unconjugated bile acids (cholic and chenodeoxycholic acids). These products will then be further modified by gut bacteria to produce secondary bile acids. A decrease in this conversion is positively correlated with liver cirrhosis. Bile acids can modulate the composition of the microbiota in the gut, where they function as signaling molecules and may constitute a mechanism of quorum sensing. In turn, the microbiota strongly affect bile acid metabolism by promoting deconjugation, dehydrogenation and dehydroxylation. It can also inhibit bile acid synthesis in the liver by alleviation of farnesoid X receptor inhibition in the ileum. Bile acids can induce FMO3 expression by an FXR-dependent mechanism.		Martin *et al.* (2007); Bennett *et al.* (2013); Gérard (2013); Kakiyama *et al.* (2013); Martínez *et al.* (2013); Sayin *et al.* (2013); Joyce *et al.* (2014)
Lipids	Cholesterol	The gut microbiota impact on the host systemic lipid metabolism. When administered as probiotics *Bifidobacteria* and *Lactobacillus* can enhance dyslipidemia and insulin resistance. Microbiota have an influence on cholesterol metabolism and weight gain in the host via the bacterial BSH mechanism.		Martin *et al.* (2007); Martínez *et al.* (2009, 2013); Yu *et al.* (2013); Joyce *et al.* (2014)
Methylamines and products of choline metabolism	Methylamine, dimethylamine, dimethylglycine, trimethylamine (TMA) and trimethylamine N-oxide (TMAO)	Cleavage of choline and phosphatidylcholine (PC) by the gut microbiota via the enzyme choline TMA-lyase produces TMA. Oxidation of TMA by hepatic flavin-containing monooxygenase 3 (FMO3) forms TMAO. Microbial metabolism of L-carnitine also produces TMA via a novel Rieske-type protein. Risk for major adverse cardiovascular events coincides with higher levels of TMAO.		Wang *et al.* (2011); Craciun and Balskus (2012); Koeth *et al.* (2013); Tang *et al.* (2013); Zhu *et al.* (2014)
Neurotransmitters	Serotonin, melatonin, glutamate, GABA, noradrenaline, dopamine and acetylcholine	It was recently discovered that gut microbiota produce tryptophan decarboxylase, the enzyme responsible for decarboxylasing tryptophan to tryptamine. Tryptamine promotes the release of serotonin by enterochromaffin cells. In a rat model it was shown that Bifidobacteria treatment resulted in increased tryptophan and kynurenic acid levels. Another study in mice showed the potential of *Lactobacillus rhamnosus* to modulate the GABAergic system. Decreased levels of dopamine were measured in fecal samples from active colitis mice.		Desbonnet *et al.* (2008); Bravo *et al.* (2011); Rooks *et al.* (2014); Williams *et al.* (2014); O'Mahony *et al.* (2015)
Phytochemicals, particularly polyphenolic compounds	Chlorogenic acids, hydrolysable tannins and flavonoids	A significant amount of polyphenols reaches the colon and is believed to contribute to gut health by promoting the growth of some commensals. Polyphenolic bioconversion by microbiota is paramount in the production of a large range of bioactive molecules. The exact roles of these molecules in health and disease are yet to be fully understood. Nonetheless epidemiological studies have tied polyphenols to health benefits such as antioxidative, anticarinogenic, antiadipogenic, antidiabetic and neuroprotective properties. Gut microbiota can also convert dietary polyphenols to benzoate.		Tomas-Barberan *et al.* (2014); Kahle *et al.* (2006); Aronov *et al.* (2011); van Duynhoven *et al.* (2011); Cardona *et al.* (2013); Marín *et al.* (2015)
Polyunsaturated fatty acids (PUFA)	Omega 3 and 6	*L. plantarum* has genes encoding for the enzyme involved in saturation metabolism of PUFA.		Kishino *et al.* (2013)
Short-chain fatty acids (SCFAs)	Most abundant acetate, propionate, butyrate; to a lesser extent—formate, fumarate, malonate, succinate, caproate and valerate	The SCFAs are produced from bacterial fermentation of non-digestible polysaccharides. They play a role in metabolic syndrome prevention and treatment. Evidence point to their potential to promote metabolic control in type 2 diabetes. SCFAs are a major source of energy for colonocytes and also contribute up to 10% of the host's daily caloric requirements. They are further involved in the control of energy utilization and maintenance of metabolic homeostasis via the G Protein coupled Receptor 43 (GPR43) receptor. SCFA products also dampen inflammatory response through this receptor. SCFAs have also been shown to affect cell proliferation and apoptosis (in cancer cells), and in epigenetic machinery such as histone acetylation by butyrate.		Bergman (1990); Maslowski *et al.* (2009); den Besten *et al.* (2013); Kimura *et al.* (2013); Natarajan and Pluznick (2014); Puddu *et al.* (2014)
Vitamins	B-group vitamins, vitamin B12; vitamin C, biotin, vitamin K	It is well established that the gut microbiota synthesize a large number of vitamins *de novo*. This is important since humans lack biosynthetic pathways for vitamins. The deleterious effects of vitamin deficiencies are well known. It has only recently been suggested that vitamin B12 may also contribute to shaping the structure and function of microbial communities in the human gut.		Hill (1997); Cooke, Behan and Costello (2006); Arumugam *et al.* (2011); LeBlanc *et al.* (2013); Degnan, Taga and Goodman (2014)
**Other noteworthy bioactives**				
Conjugated linoleic acid (CLA), bacteriocin		CLA is associated with a diverse array of biological activities, and predominantly associated with activation of peroxisome proliferator activated receptors (PPARs) and the associated switching on and off of genes. Some Bifidobacteria and *Lactobacillus* species have been shown to produce CLA. Bacteriocins are peptides synthesized by bacteria and have narrow (same species) or broad (across genera) spectrum activity against other bacteria. A large number of archaea and bacteria are believed to produce at least one bacteriocin.		Bowdish, Davidson and Hancock (2005); Ross *et al.* (2010)
Tetrathionate and nitric oxide		Tetrathionate and nitric oxide are produced in an inflammatory environment and are central to the fitness of several Enterobacteriaceae. Tetrathionate utilization positively correlated with active colitis in a mouse model. Bacterial growth depends on the presence of nitrogen. Synthesis of amino acids by the microbiome depends on the recycling of nitrogen back into gastrointestinal organs.		Winter *et al.* (2010); Bergen (2014); Rooks *et al.* (2014)

By contrast, our theme here is that it is additionally the microbes themselves that can pass from the gut (and other ‘external’ surfaces) into the human body, a phenomenon sometimes known as ‘dysbiosis’, albeit this term is more commonly used with another meaning. We here need to discriminate a changed (pathologic) microbiota in the place of origin from the results of a translocation of microbiota to other areas of the body. In the following sections, we use the term dysbiosis to describe changes in a microbiome in its main origin (typically the gut), and we coin the term ‘atopobiosis’ to describe microbes that appear in places other than where they should be.

### The origin of detectable but non-proliferating microbes appears to be mainly via ‘atopobiosis’ of the gut microbiome

Dysbiosis, also known as dysbacteriosis, particularly referring to microbial imbalance in the digestive tract, has been widely discussed (e.g. Scher and Abramson 2011; Scanlan *et al.* 2012; Amar *et al.* 2013; Bested, Logan and Selhub 2013; Duytschaever *et al.* 2013; Vaarala 2013). Core to this literature is the idea that factors that lead to significant changes in the gut microbiota composition (dysbiosis) ultimately result in pathology (Larsen *et al.* 2010; Amar *et al.* 2011, 2013; Bested, Logan and Selhub 2013; Burcelin *et al.* 2013; De Angelis *et al.* 2013; Fremont *et al.* 2013; Lanter, Sauer and Davies 2014; Petriz *et al.* 2014; Power *et al.* 2014; Tojo *et al.* 2014). Table 3 gives a list of diseases, largely inflammatory diseases, which have been associated with gut dysbiosis.

**Table 3.** Various pathologies that have been associated with dysbiosis of the gut.

Condition	References
Asthma	Abrahamsson *et al.* (2014)
AD	Karri, Martinez and Coimbatore (2010); Alam *et al.* (2014)
Atherosclerosis	Koren *et al.* (2011)
Autism spectrum disorders	Parracho *et al.* (2005); Finegold *et al.* (2010); Adams *et al.* (2011); Williams *et al.* (2011, 2012); De Angelis *et al.* (2013); Kang *et al.* (2013)
β-Cell autoimmunity	de Goffau *et al.* (2014)
Cardiovascular disease	Amar *et al.* (2011)
Crohn's disease	Seksik *et al.* (2003)
Chronic fatigue syndrome	Sheedy *et al.* (2009); Proal *et al.* (2013)
Cystic fibrosis	Scanlan *et al.* (2012); Bruzzese *et al.* (2014) ;Sánchez-Calvo *et al.* (2008); Duytschaever *et al.* (2011, 2013); Madan *et al.* (2012)
HIV/AIDS	Lozupone *et al.* (2013); McHardy *et al.* (2013); Vujkovic-Cvijin *et al.* (2013)
IgE-associated eczema	Abrahamsson *et al.* (2012)
Inflammation	Cani *et al.* (2008, 2012); Delzenne and Cani (2011); Delzenne *et al.* (2011)
Inflammatory bowel disease	Conte *et al.* (2006); Clemente *et al.* (2012); Manichanh *et al.* (2012); Morgan *et al.* (2012); Nagalingam and Lynch (2012); Bakhtiar *et al.* (2013)
Iron deficiency	Balamurugan *et al.* (2010); Zimmermann *et al.* (2010); Dostal *et al.* (2012, 2014)
Liver disease	Schnabl and Brenner (2014)
Multiple sclerosis	Berer *et al.* (2011)
Obesity	Delzenne and Cani (2011); Geurts *et al.* (2014)
Rheumatoid arthritis	Detert *et al.* (2010); Berer *et al.* (2011); Scher and Abramson (2011); Bingham and Moni (2013); Brusca, Abramson and Scher (2014); Catrina, Deane and Scher (2014); Cénit *et al.* (2014); Demoruelle, Deane and Holers (2014); Taneja (2014)
Parkinson's Disease	Scheperjans *et al.* (2015); Vizcarra *et al.* (2015)
Sarcoidosis	Almenoff *et al.* (1996)
Systemic lupus erythematosus	Hevia *et al.* (2014); Zhang *et al.* (2014a)
Symptomatic atherosclerosis/stroke	Karlsson *et al.* (2012)
Type 1 diabetes	Brown *et al.* (2012); Owen and Mohamadzadeh (2013); Petersen and Round (2014)
Type 2 diabetes	Larsen *et al.* (2010); Brown *et al.* (2012); Qin *et al.* (2012); Karlsson *et al.* (2013); Everard *et al.* (2014)

**Figure 3.**
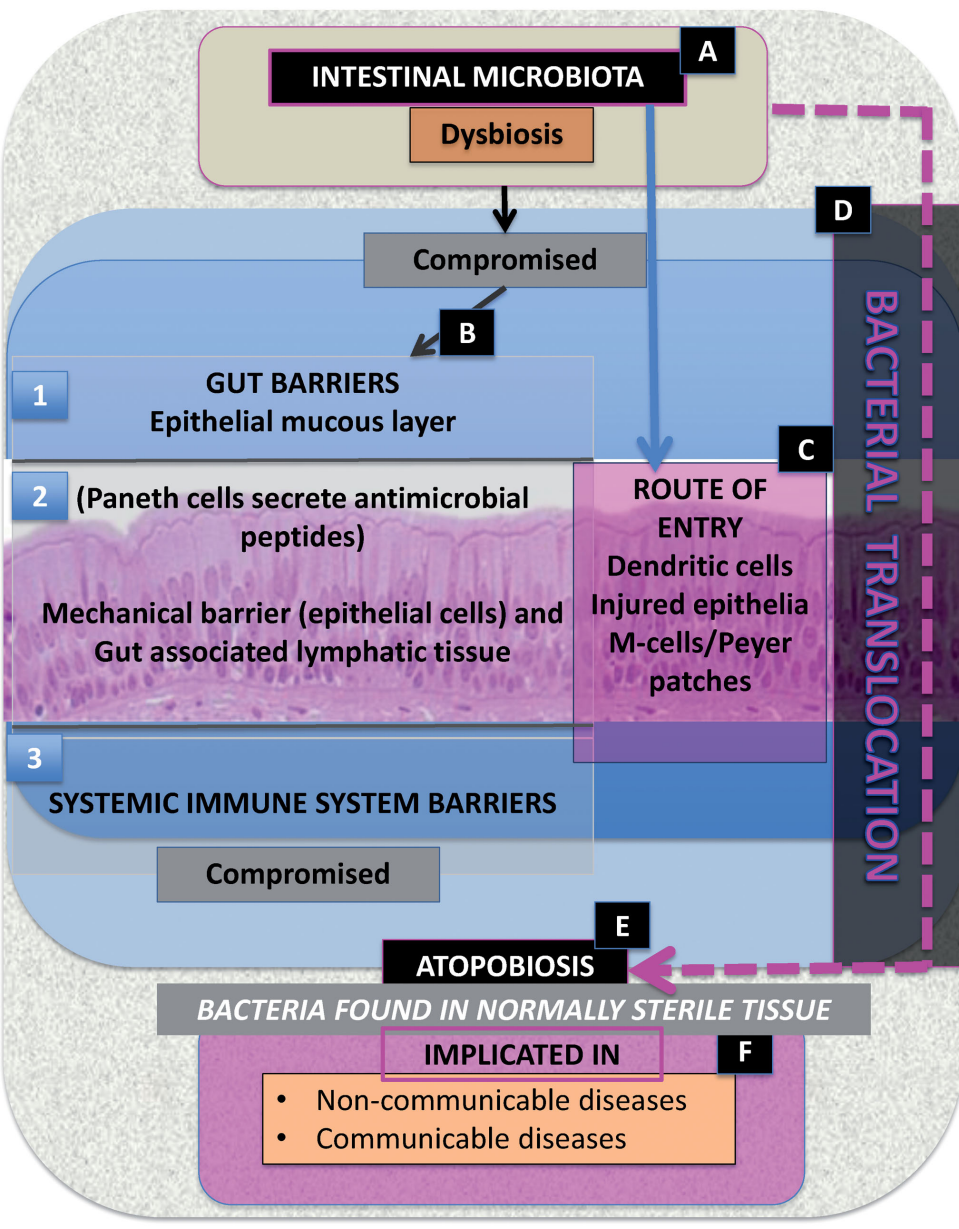
Schematic representation of dysbiosis, bacterial translocation and atopobiosis. (**A**) When intestinal microbiota are associated with dysbiosis, (**B**) the gut barrier (1 and 2) becomes compromised; this leads to (**C**), a route of entry via the gut epithelia causing (**D**) bacterial translocation. Bacterial translocation is also associated with a compromised systemic immune system barrier (3). Therefore, intestinal microbiota dysbiosis (A) followed by bacterial translocation (D) results in (**E**) atopobiosis. (**F**) The results of bacterial translocation are seen in various conditions (see Table 4).

In addition, we argue here that as well as gut dysbiosis, a derangement of the gut microbiome, what we are seeing here, often called ‘translocation’ in the context of surgery (Swank and Deitch 1996; MacFie 2004) and various diseases (Berg 1995) (see Table 4 that lists diseases and conditions where bacterial translocation is specifically implicated), is what might better be called atopobiosis (Greek ατoπoς or atopos, in the wrong place), i.e. an appearance of members of the gut (or other) microbiome in the wrong place. Bacterial translocation is therefore discussed in the context of the movement of gut origin microbes [that changed from normal (dysbiosis)] that moved across the ‘intact’ gastrointestinal tract into normally sterile tissues, including blood, where the organisms may then directly cause infection or inflammation leading to tissue injury, organ failure, etc. (Steinberg 2003; Wiest and Rath 2003; Balzan *et al.* 2007). We stress that they may be found in both infectious and non-infectious diseases as well as being translocated during surgery, and that atopobiosis of bacteria originating in the oral cavity, e.g. in periodontal disease, may also be significant in rheumatoid arthritis, for instance (see below). Fig. 3 provides a schematic representation of dysbiosis, bacterial translocation and atopobiosis.

**Table 4.** Diseases and conditions where bacterial translocation (of gut or oral origin) and consequent chronic infection are specifically implicated

Diseases and conditions where translocation of bacteria are present	References
**Communicable diseases**	
Fibrosis stage in HIV/HCV coinfection	Balagopal *et al.* (2008); Montes-de-Oca *et al.* (2011); Page, Nelson and Kelleher (2011); Lin, Weinberg and Chung (2013); Sacchi *et al.* (2015)
Hepatitis C virus (HCV) infection	French *et al.* (2013); Munteanu *et al.* (2014)
HIV/AIDS infection	Sandler and Douek (2012); Klatt, Funderburg and Brenchley (2013); Vázquez-Castellanos *et al.* (2014)
Pneumonia in immunocompromised patients	Sawa (2014)
**Diseases usually seen as non-communicable**	
Abdominal compartment syndrome	Mifkovic *et al.* (2013)
Alcoholic liver disease	Chen and Schnabl (2014); Malaguarnera *et al.* (2014)
Allergic disease: bacterial translocation during pregnancy	Abrahamsson Wu and Jenmalm (2015)
Atherosclerosis	Epstein, Zhou and Zhu (1999); Kozarov *et al.* (2006); Erridge (2008); Renko *et al.* (2008); Epstein *et al.* (2009); Nagata, de Toledo and Oho (2011); Rosenfeld and Campbell (2011); Hopkins (2013); Dinakaran *et al.* (2014); Rogler and Rosano (2014); Trøseid *et al.* (2014)
Burn wounds	Macintire and Bellhorn (2002); Sharma (2007); Aboelatta *et al.* (2013)
Cirrhosis	Wiest and Garcia-Tsao (2005); Jun *et al.* (2010); Giannelli *et al.* (2014); Wiest, Lawson and Geuking (2014)
Chronic kidney disease	Anders, Andersen and Stecher (2013); Sabatino *et al.* (2014)
Metabolic syndrome	Festi *et al.* (2014)
Non-alcoholic fatty liver disease	Bieghs and Trautwein (2014)
Obesity	Vajro, Paolella and Fasano (2013); Sanz and Moya-Pérez (2014)
Pancreatitis	Mifkovic *et al.* (2009); Guo *et al.* (2014); Oláh and Romics (2014)
Rheumatoid arthritis	Ogrendik (2009b, 2013b); Ebringer and Rashid (2014); Koziel, Mydel and Potempa (2014)
Schizophrenia	Severance *et al.* (2013); Severance, Yolken and Eaton (2014)
Sepsis and Septic shock*	Tsujimoto, Ono and Mochizuki (2009); Wallet *et al.* (2011); Deitch (2012); Leli *et al.* (2014)
Stroke	Syrjänen *et al.* (1988); Emsley and Tyrrell (2002); Emsley *et al.* (2003); Emsley and Hopkins (2008); McColl, Allan and Rothwell (2009); Emsley and Chamorro (2010); Grau, Urbanek and Palm (2010); Wang *et al.* (2012a); Chien *et al.* (2013); Dalager-Pedersen *et al.* (2014); Fugate *et al.* (2014)
**Surgical procedures**	
Bariatric surgery	Festi *et al.* (2014)
Cardiac surgery	Allen (2014)
Multiple organ failure (MOF)	Swank and Deitch (1996)
Sepsis due to surgery	MacFie (2004); Puleo *et al.* (2011)

*‘Sepsis’ is widely used to imply living microbes, but as is now well known it can also occur in the absence of any culturable microbes, including those incapable of proliferation due to antibiotic activity. Sepsis may commonly result simply from the effects of molecules such as LPS on the generation of inflammatory cytokines (Kotsaki and Giamarellos-Bourboulis 2012; Balakrishnan *et al.* 2013).

### How do gut bacteria escape into blood?

If the gut microbiome is seen as the main source of the blood microbiome, it is necessary to establish which kinds of conditions might permit this in the absence of real physical damage (as may, for instance, be caused by surgery) leading to microbial translocation. Wiest, Lawson and Geuking (2014) mention three possible points of entrance for bacteria into the surrounding (sterile) tissue:

- by dendritic cells via processes between epithelial cells, not affecting tight junction function,
- via injured/inflamed epithelium with dysfunctional epithelial barrier,
- and via M cells overlying Peyer's patches as specialized cells providing access of microbial products to antigen-presenting cells.

We discuss bacterial translocation in this context in the following sections.

### The role of M cells and Peyer's patches in gut microbial translocation and atopobiosis

While the gut epithelium represents the largest mucosal tissue, the mechanisms underlying the interaction between the microbiome and the epithelial cells remain poorly understood (Mathias *et al.* 2014). Although this is a vast and complex field that warrants a review of its own, we briefly argue that gut dysbiosis results in an atypical interaction of both the microbiota, as well as their secretory products, with the gut epithelial layer. This results in an altered barrier function, which may also lead to changed mucosal immunity and ultimately to atopobiosis. The gut epithelium is necessarily normally quite impermeable to microbes, but there is increasing evidence that direct chemical communication between the microbiota and the epithelial cells regulates mucosal integrity (Venkatesh *et al.* 2014). A possible point of entry is by direct cellular uptake, and there is one type of cell that can take up microbes, and these are the M cells overlaying the Peyer's patches (Kernéis *et al.* 1997; Jepson and Clark 1998; Clark and Jepson 2003; Corr, Gahan and Hill 2008; Lelouard *et al.* 2010; Fukuda, Hase and Ohno 2011). Peyer's patches are seen as the ‘immune sensors’ of the gut epithelium. Considerable evidence exists that they provide a primary route for the limited translocation of microbes between the gut epithelium and the blood system (Jung, Hugot and Barreau 2010). These interactions with the cells of the gut may suggest that changes in the intestinal microbiota also influence mucosal immunity (Sato, Kiyono and Fujihashi *et al.* 2014). This is indeed the case, and gut dysbiosis has been shown to play a significant role in the development of autoimmune diseases, in particular inflammatory bowel diseases (Clemente *et al.* 2012; Morgan *et al.* 2012; Hold *et al.* 2014; Kostic, Xavier and Gevers 2014; Owyang and Wu 2014; Ma *et al.* 2015). It was also suggested that a changed gut microbiota represents the initial site of autoimmunity generation, and might be a critical epigenetic factor in autoimmune diseases such as rheumatoid arthritis (Scher and Abramson 2011; Luckey *et al.* 2013; Brusca, Abramson and Scher 2014; Catrina, Deane and Scher 2014; Cénit *et al.* 2014; Taneja 2014). There is also evidence that regulatory T cells in the gut are influenced by microbial factors, and that a changed microbiota (dysbiosis) may influence the induction and suppressor functions of these cells, in turn leading to a changed gut mucosal immunity (Kinoshita and Takeda 2014).

We have earlier reviewed the literature that suggests that dysbiosis can cause gut epithelial barrier dysfunction, and thereby provide a point of entry into the body, including the blood, resulting in atopobiosis. This is supported by recent research that has suggested that blood microbiota might be implicated in various (cardiovascular and other) diseases. Sequence-based techniques provided evidence for the presence of such a blood microbiome. The question now arises as to whether such a microbiome's presence can be directly measured by e.g. ultrastructural (microscopic) methods, since a consequence of any translocation of microbes between the gut microbiome and blood is that they should then be observable in blood. The next sections will provide visual evidence of the presence of such a microbiota in Alzheimer's disease (AD) and PD. As shown in Table 3, these conditions are known to be associated with the presence of dysbiosis.

### Direct measurement by ultrastructural (microscopic) methods

**Figure 4.**
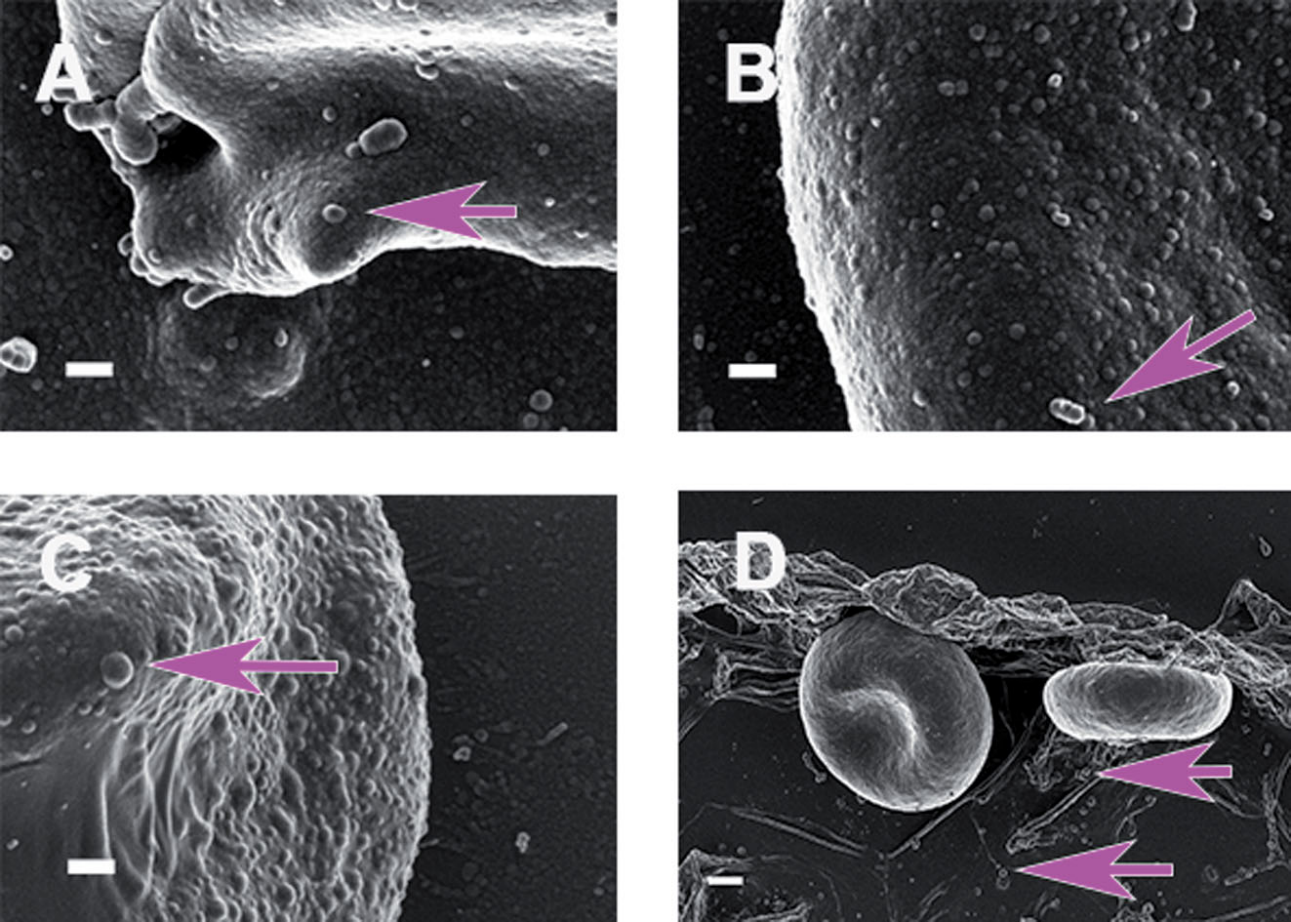
Micrographs taken from previously published manuscripts. (**A**–**C**) Bacterial presence in PD, originally shown in Fig. 8A, C and **G** in Pretorius *et al.* (2014a). (**D**) Bacterial presence in AD, originally shown in Fig. 2 in Lipinski and Pretorius (2013).

Direct measurement by ultrastructural (microscopic) methods of analysis shows that microbes are in fact common constituents of blood in inflammatory diseases [previously seen in PD—Fig. 8 in (Pretorius *et al.* 2014a and in AD—Fig. 2 in (Lipinski and Pretorius 2013). We show and annotate selected micrographs from these papers in Fig. 4]. An important concern that needs to be addressed, as is also the case with sequence-based methods, is whether the presence of microbiota in whole blood is indeed not the result of introduced external contamination. There is in fact considerable evidence in the literature that bacteria as well as other microorganisms can reside inside RBCs (e.g. Minasyan 2014), and thus able to cross the RBC membrane somehow (see Table 5). Transmission electron microscopy (TEM) analysis showing bacteria inside cells would also tend to imply that the bacteria were not externally introduced artefactually during the preparation of the samples.

**Table 5.** Some microorganisms that are known to invade red blood cells.

Pathogen	Type of microorganism	Mechanism of invasion	References
*Anaplasma marginale*	A tick-borne pathogen that causes the disease anaplasmosis in cattle.	Via major surface protein 1a (MSP1a)	Kocan *et al.* (2004)
*Bartonella bacilliformis* *B.quintana*	*Bartonella* species are fastidious Gram-negative bacteria, which belong to the alpha group of the domain Proteobacteria.	The Trw T4SS mediates attachment of *Bartonella* to red blood cells in *Bartonella* lineage 4. *Bartonella* is collected in pits and trenches that form as a result of deformation factor. Invaginations supposedly pinch off to carry the content in a vacuole structure to the cytoplasm of the red blood cell where the organism persists.	Iwaki-Egawa and Ihler (1997); Coleman and Minnick (2001); Rolain *et al.* (2003); Eicher and Dehio (2012)
*Brucella melitensis*	Facultative intracellular Gram-negative coccobacilli.	Invasion shown in mouse erythrocytes. Mechanism to be identified.	Vitry *et al.* (2014)
*Francisella tularensis*	Highly infectious bacterium, which can cause severe disease tularemia with an infection of fewer than 10 bacteria	Via serum complement-dependent and independent mechanisms.	Conlan (2011); Horzempa *et al.* (2011)
*Mycoplasma suis*	A member or the hemotrophic mycoplasma group that parasitize erythrocytes in pigs.	Invasion occurs in a similar manner to that of *P. falciparum* and *B. bacilliformis*. Attachment via MSG1 (GAPDH) protein.	Groebel *et al.* (2009); Zhang *et al.* (2014c)
*M. bovis*	Small cell wall-less bacterium that contributes to a number of chronic inflammatory diseases in dairy and feedlot cattle.	Undetermined.	van der Merwe, Prysliak and Perez-Casal (2010)
*M. gallisepticum*	Mycoplasmas are small cell wall-less prokaryotes.	Not known.	Vogl *et al.* (2008)
*Plasmodium falciparum*	The main malaria parasite, part of whose life cycle involves inhabiting RBCs.	Recognition of surface receptors precedes a reorientation where the apical end is adjusted to the erythrocyte. A tight junction that involves high-affinity ligand receptor interactions is formed. The tight junction moves from the apical to posterior pole and is powered by the actin-myosin motor of the parasite. The adhesive proteins at the junction are proteolytically removed when the posterior pole is reached, most likely by a rhomboid resident protease in a process that facilitates membrane resealing. The invasion process produces a parasitophorous vacuole containing the merozoite.	Cowman and Crabb (2006)
*Streptococcus pneumoniae*	Gram-positive bacterium which causes infection-related diseases.	LPXTG motif-containing pneumococcal proteins, erythrocyte lipid rafts and erythrocyte actin remodeling are involved in the invasion mechanism.	Yamaguchi *et al.* (2013)
*Theileria sporozites*	Intracellular protozoan transmitted by ixodid ticks. Infect wild and domesticated ruminants. Phylogenetically most closely related to *Babesia*.	Occurs in a similar manner to sporozoite entry.	Shaw (2003); Bishop *et al.* (2004)

**Figure 5.**
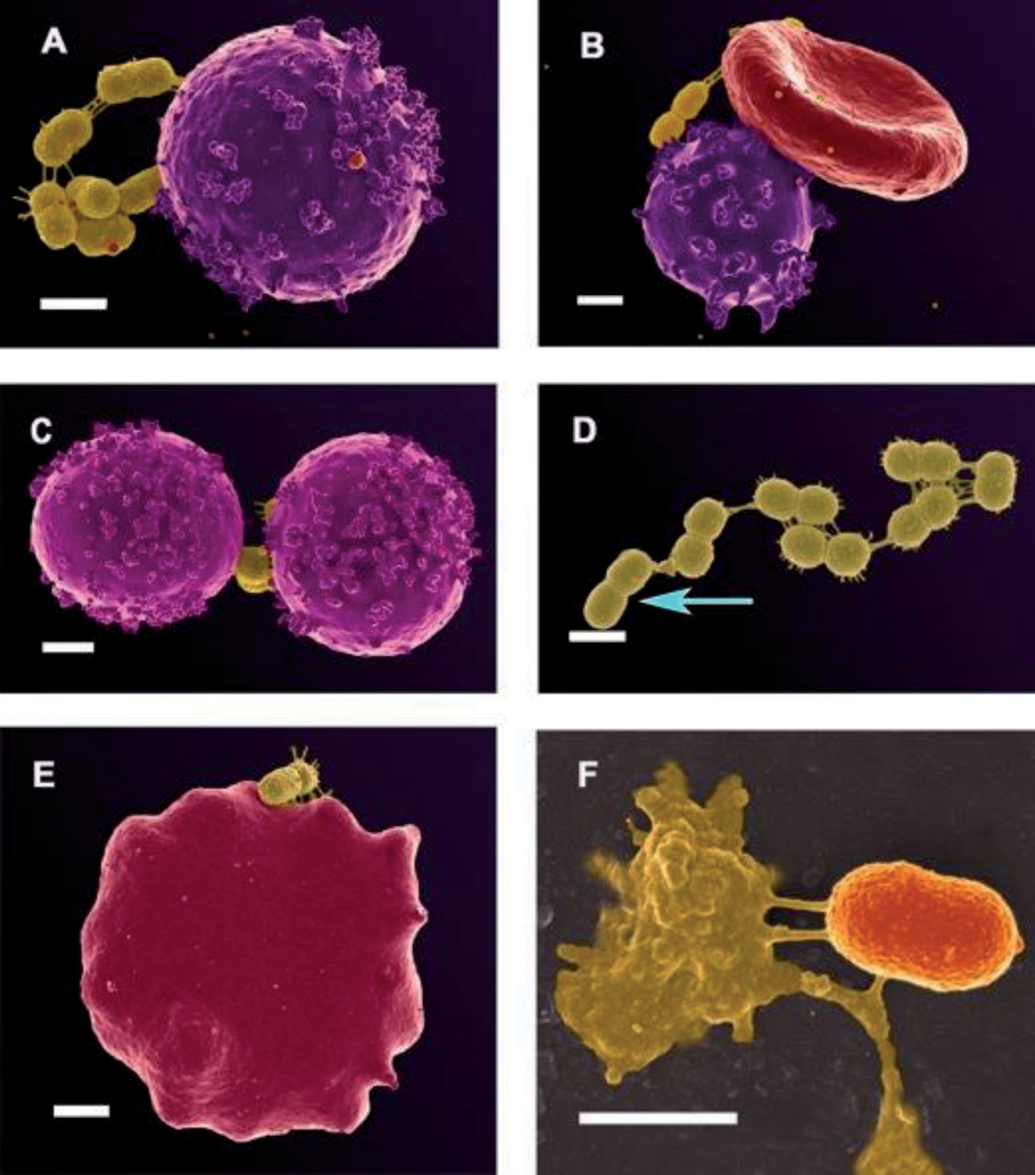
RBCs with microbiota from patients with diagnosed AD (additional micrographs from sample used in Lipinski and Pretorius 2013). These micrographs are representative of bacteria found in smears of 14 of the 30 AD individuals. (**A** and **B**) coccus-shaped bacteria associated with white blood cell; (**B**) coccus-shaped bacteria associated with an erythrocyte and white blood cell; (**C**) two white blood cells associated with coccus-shaped bacteria; (**D**) a string of cocci-blue arrow shows possibly dividing coccoid bacteria; (**E**) an erythrocyte associated with coccus-shaped bacteria; (**F**) a high machine magnification of a coccus-shaped bacteria associated with a dense matted fibrin deposit. Scale bar: 1 μm.

**Figure 6.**
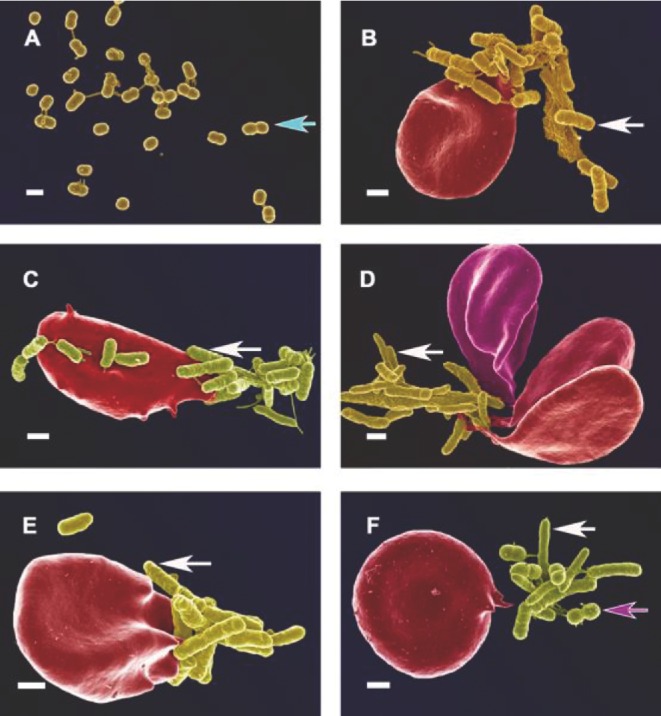
RBCs with microbiota from patients with diagnosed PD (additional micrographs from sample used in Pretorius *et al.* 2014a). These micrographs are representative of bacteria found in smears of 21 of the 30 PD individuals. (**A**) A collection of coccus- and bacillus-shaped bacteria; (**B**) coccus- and bacillus-shaped bacteria associated with erythrocyte; (**C**) bacillus-shaped bacteria in close proximity with erythrocyte. Erythrocyte forms extensions towards bacteria; (**D** and **E**) bacillus-shaped bacteria associated with elongated erythrocytes; (**F**) coccus- and bacillus-shaped bacteria close to erythrocyte that extends pseudopodia towards the bacteria. Coccus-shaped bacteria shown with pink arrows; bacillus-shaped bacteria shown with white arrows. Dividing coccus-shaped bacteria shown with blue arrow. Scale bar: 1 μm.

For the current paper, we have revisited our AD and PD samples and figures from Pretorius *et al.* (2014a) and Lipinski and Pretorius (2013) and noted the prevalence of bacteria in almost all of the AD and PD samples, in numbers much in excess of those seen in our database of thousands micrographs from healthy individuals. Here we show additional micrographs from the previously published samples (see Figs 5 and 6). In both conditions (see Figs 5AD and 6PD), microbes were noted in close proximity to RBCs, and in some cases RBCs extended pseudopodia-like projections towards the microbiota. SEM analysis of AD whole blood (Fig. 5) shows that mostly coccus-shaped bacteria are present. White blood cells are seen in close proximity to these bacteria in AD patients (see Fig. 5A–C). SEM analyses of PD patients (Fig. 6) show both coccus- and bacillus-shaped bacteria in close proximity to RBCs. We also observed that RBCs extend pseudopodia towards these bacteria and this might be part of the mechanism by which the bacteria enter the RBCs (see Fig. 6C–F). We also note possibly dividing coccus-shaped bacteria in both these conditions, indicated with blue arrows on Fig. 5A (AD patient) and Fig. 6D (PD patient). This might suggest that these bacteria may be(come) culturable under appropriate conditions (see also Soina *et al.* 2012; Epstein 2013).

**Figure 7.**
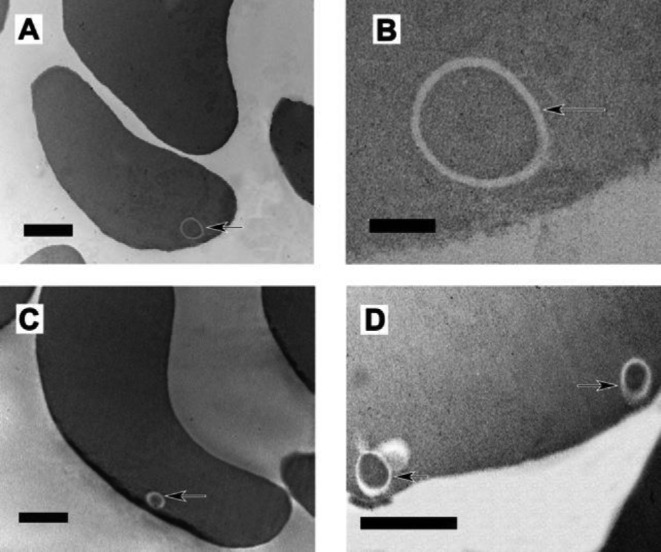
TEM confirming the presence of bacteria inside erythrocytes of (**A** and **B**) AD, (**C** and **D**) PD. (Additional micrographs from sample used in Lipinski and Pretorius (2013) and Pretorius *et al.* (2014a). Arrows in each micrograph show the presence of cellular inclusions, without visible membranes. Inclusions are not typically noted in erythrocytes. We suggest that these inclusions are bacteria, possibly as L-forms. Scale bar = 1 μm (A, C, D); 200 nm (D).

TEM analysis of the samples from Lipinski and Pretorius (2013) and Pretorius *et al.* (2014a) showed the presence inside RBCs of cells that appeared to be microbial in nature (unpublished data). These internalized cells further provide evidence for a sustained presence of such a blood microbiota (and one hardly explained by contamination) (see Fig. 7A and B: AD and C and D: PD). Bacteria are shown with arrows in the micrographs. No bacterial membrane was noted; therefore, the bacteria may be L-forms. There seems to be bacterial species selectivity for a given disease, as our preliminary observations suggest a prevalence for bacillus-type bacteria in AD, but both coccus- and bacillus-shaped bacteria in PD patients.

Our observations suggest that the presence of bacteria in these two diseases occurs in only a small fraction of the RBC population, which is why we had not really noted them in our previous studies (e.g. Bester *et al.* 2013; Pretorius *et al.* 2013, 2014a,b; Pretorius and Kell 2014), and SEM and TEM analysis confirms this observation. We have never (or not yet) found bacteria inside RBCs from healthy controls (these without overt, diagnosed diseases) when studying blood smears using TEM analysis. The microscopy preparation methods involve a washing process, and this may wash away some of the bacteria, or RBCs and white blood cells associated with bacteria. Therefore, the actual quantification of the bacteria can only be done by other means; however, dormancy and viability versus non-viability issues pertain (as discussed above).

We found a definite association between RBCs and bacteria, with RBCs (see Figs 6 and 7) forming pseudopodia-like extension, as if in the process of engulfing bacteria. Both coccoid (round) and bacillary (elongated) bacteria were found in PD whole blood SEM micrographs, but only coccoid forms in AD whole blood SEM micrographs. Samples from 25 diagnosed AD patients were studied and bacteria were detected in 14 individuals from this AD sample, while samples from 30 PD patients were studied, in 21 of whom we detected bacteria. Obviously, the type of bacteria cannot be identified from ultrastructural observations. As with the timeline of established cases such as the role of *H. pylori* in ulcers and colon cancer, the next tasks are to bring these microscopically observed bacteria into culture and to carry out sequence-based studies to establish their role (if any) in non-communicable diseases. However, to illustrate that the bacteria may indeed be engulfed by the RBCs, and to confirm that the phenomenon is not due to external contamination, we show TEM micrographs from both of the studied diseases (see Fig. 7, AD and PD).

## CONCLUDING REMARKS AND PROSPECTIVE EXPERIMENTS

‘Non-culturable’ (which should be called ‘not-easily-culturable’ or ‘not-yet-cultured’) microbes are commonplace in the ‘environmental microbiology’ of soil and water, and the blood certainly represents an ‘environment’. As we show here, there is a large and scattered literature, increasing in size, to the effect that there might be a (mainly dormant) microbial component in a variety of chronic diseases that are normally considered to be non-microbial or non-communicable in nature, even when microbes appear absent by culturability criteria. Our previous work (e.g. Bester *et al.* 2013; Pretorius *et al.* 2013, 2014a; Kell and Pretorius 2014, 2015; Pretorius and Kell 2014) has implied iron dysregulation as a regular accompaniment to, and probable contributory factor for, a variety of similar diseases, all of which have an inflammatory component. We argue here that there is also a microbial contribution to this in the blood, and it is not unreasonable that the microbial requirement for iron means that, despite the oxidative stress it can entail (Touati 2000; Kell 2009, 2010), microbes may be anticipated to increase in prevalence when iron is free (e.g. Ratledge 2007; Clifton, Corrent and Strong 2009; Sia, Allred and Raymond 2013; Chu *et al.* 2014) and available (D'Onofrio *et al.* 2010), probably behaving in a social manner (Kell, Kaprelyants and Grafen 1995; West and Buckling 2003; Diggle *et al.* 2007; Harrison and Buckling 2009).

**Figure 8.**
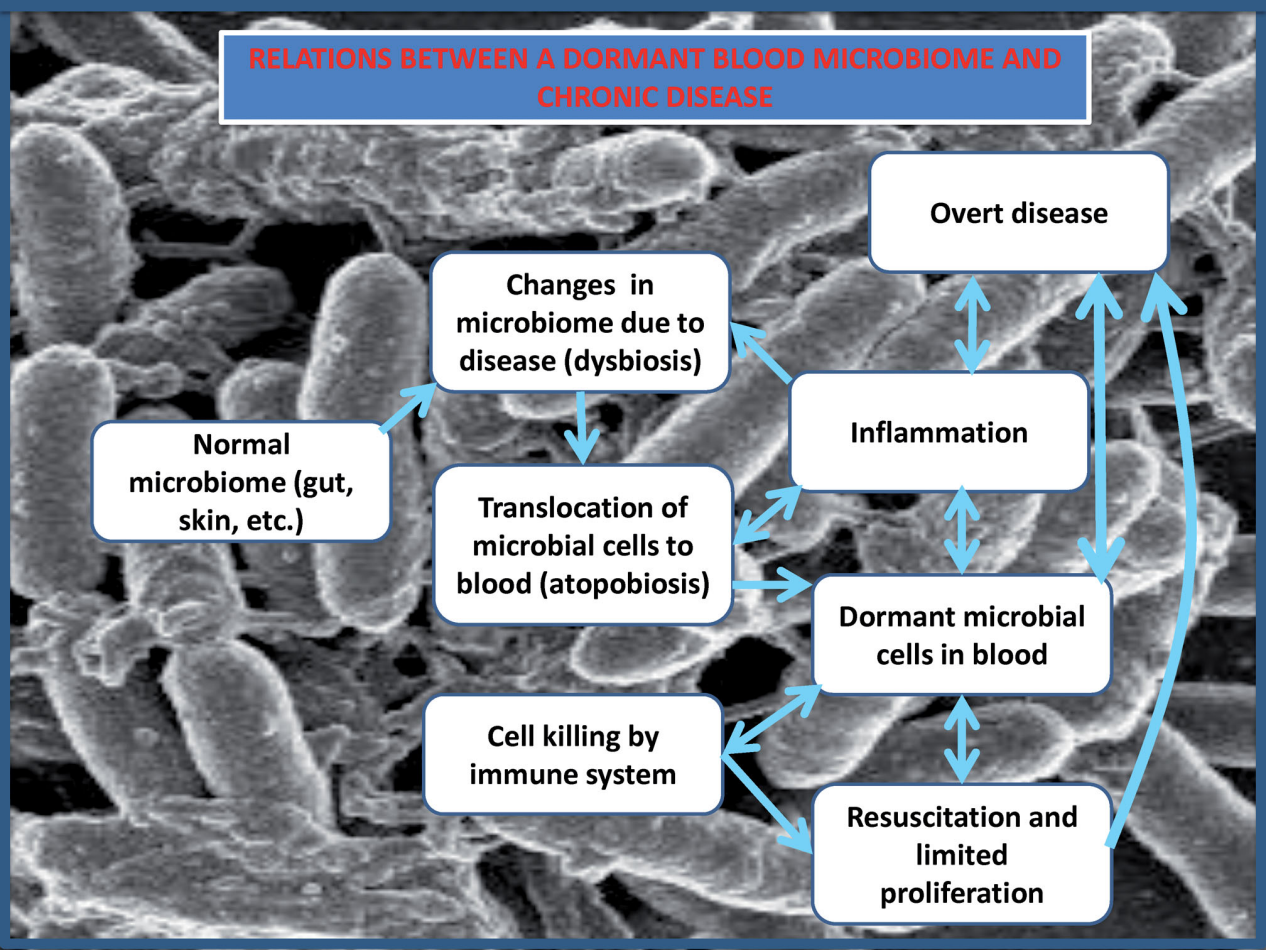
Relationships between a dormant blood microbiome and chronic disease dyamics.

We have here pointed up the likelihood of a steady crop of effectively dormant microbes being a feature of blood biology in chronically diseased humans, including those with non-communicable diseases. As with any complex system, the magnitude of any component is affected by the kinetics of every relevant step; while the precise nature of all the interactions is uncertain, Fig. 8 describes the general network—the first step in any systems analysis (Kell 2006; Kell and Knowles 2006).

Consequently, we recognize that the analysis above has largely been qualitative (the ‘presence’ of a microbial component in a specific disease is a qualitative statement). However, chronic, non-communicable diseases are very far from being static (and thousands of human genes change their expression at least 2-fold even on a diurnal basis; Zhang *et al.* 2014b). Thus, a clear further issue is to seek to understand how the blood microbiome may co-vary with the day-to-day dynamics of chronic diseases. For example, rheumatoid arthritis has circadian rhythms (Straub and Cutolo 2007) and is well known to provide significant variations (‘flares’; Flurey *et al.* 2014) in severity at different times. A reasonable strategy is thus to look for changes in a detectable blood microbiome in this and other diseases that show such flares. As with *H. pylori* and stomach ulcers (and cancer), the simple prediction is that bactericidal antibiotics should be of value in the treatment of such supposedly non-communicable diseases. Indeed, this prediction is borne out for diseases such as rheumatoid arthritis (Ogrendik 2009a, 2013a; Kwiatkowska and Maślińska 2012) and multiple sclerosis (Ochoa-Repáraz *et al.* 2009; 2011), while antipneumococcal vaccination has shown efficacy in preventing stroke (Vila-Corcoles *et al.* 2014). Of course, events such as heart attacks and strokes (and see Table 4) may also be seen as sudden increases in severity of an underlying condition, and in some cases (such as the much increased likelihood of strokes after subarachnoid haemorrhages; McMahon *et al.* 2013), analysis of changes in the blood microbiome might prove predictive.

The obvious next tasks are thus to relate the number and nature of blood microbes observed in cases such as the above to microbial sequences and antigens that can be detected in aliquots of the same samples (e.g. Salipante *et al.* 2013, 2015), to determine the physiological state of the various microbes (including e.g. whether they are L-forms), and to establish methods to bring them (back) into culture. Since microbes, inflammation and various syndromes are such common co-occurrences (as are coagulopathies; Kell and Pretorius 2015), longitudinal studies will have a specially important role, as they will both show the dynamics and be able to help discriminate cause and effect during the time evolution of chronic, non-communicable diseases in ageing populations. The immunogenicity of persisters, and their ability to induce various kinds of inflammation, must be rather different from that of replicating organisms, and this must be investigated. Armed with such collective knowledge, we might be better placed to develop therapeutics such as pre- and probiotics and bactericidal antibiotics for use in such cases previously thought to lack a microbial contribution.

## FUNDING

We thank the Biotechnology and Biological Sciences Research Council (grant BB/L025752/1) as well as the National Research Foundation (NRF) of South Africa for supporting this collaboration.

***Conflict of interest.*** None declared.

## GLOSSARY

16S ribosomal RNA: a component of the 30S small subunit of prokaryotic ribosomes. The 16S rRNA gene is found in all bacteria and archaea and consists of nine short hypervariable regions that may be used to distinguish bacterial taxa.

Anabiosis: when an organism is in a state of very low metabolic activity to the extent where it is hardly measurable and in some cases come to a standstill. The physiological and biochemical processes are arrested for different periods of time but can be reversed.

Atopobiosis (Greek $\mathop {\rm \alpha }\limits^{,{\rm \prime }} {\rm \tau oo\pi o}\varsigma$ or atopos) appearance of the gut or other microbiome in the wrong place.

Bacterial translocation: the passage of viable resident bacteria from the gastrointestinal tract to normally sterile tissues such as the mesenteric lymph nodes and the other internal organs.

Cryptobiosis: refers to latent life or a state where an organism lacks any visible signs of life but is not dead in that it may revert to a state of aliveness as usually defined. Its metabolic activity becomes hardly measurable, or comes reversibly to a standstill.

Culturability: the ability of a cell to reproduce.

Direct viable count: the original method comprises incubation of samples with nutrients (yeast extract) and a single antimicrobial agent that specifically inhibits DNA synthesis but not RNA synthesis (nalidixic acid). Cell division ceases as a result of DNA synthesis inhibition but other cellular metabolic activities remain unaffected and therefore cells continue to metabolize nutrients and grow in size, which allows their detection microscopically *in situ*.

Dormant: not viable in the sense of not being more or less immediately culturable, but may be returned to a state of viability or culturability by preincubation under suitable conditions.

Dysbiosis: derangement of the species distribution in the normal microbiome.

L-forms: these bacteria are cell wall-deficient forms of normal bacteria. They are able to proliferate as sphaeroplasts or protoplasts under certain conditions.

Metagenomics: direct genetic analysis of a collection of genomes contained in an environmental sample.

Microbiome: the genetic sum of the ecological community of commensal, symbiotic and pathogenic microorganisms that lives on and inside our bodies.

‘Most Probable Number’ technique: is a method used to quantify the concentration of viable microorganisms in a sample. It involves replicate liquid broth growth in 10-fold dilutions. When a dilution lacks growth, it is assumed not to have any organisms. Back-calculation via a Poissonian distribution leads to the ‘most probable number’ in the original sample

Non-axenic culture: contains more than one species, variety or strain of organism.

Non-viable: incapable of observable replication by any stated means normally capable of effecting replication in the relevant organism.

Phylogenetics: a discipline of evolutionary biology that studies the relationships between organisms based on how closely similar some of their macromolecular sequences are.

Pleomorphic: possessing the ability to change shape or size in response to environmental stimuli.

Resuscitation: induction of apparently non-culturable cells to a state of culturability.

Sterile: refers to an absence of operationally viable organisms.

Viable: capable of observable replication, i.e. culturable, by any stated means.
